# Effect of vitamin C supplementation on outcomes in patients with COVID-19: a systematic review and meta-analysis

**DOI:** 10.3389/fnut.2024.1465670

**Published:** 2024-10-03

**Authors:** Wenhao Xu, Peng Wang, Jun Wan, Yaheng Tan, Yuyang Liu, Qiwen Chen, Yuxin Zheng, Xueying Yu, Sitong Fan, Cuyubamba Dominguez Jorge Luis, Yu Zhang

**Affiliations:** ^1^Center for Evidence-based Medicine, Affiliated Hospital of Chengdu University, Chengdu, China; ^2^Department of Critical Care Medicine, Affiliated Hospital of Chengdu University, Chengdu, China; ^3^West China Hospital, Sichuan University, Chengdu, China

**Keywords:** vitamin C, COVID-19, meta-analysis, hospital mortality, length of stay

## Abstract

**Background:**

Since the emergence of the coronavirus disease 2019 (COVID-19) pandemic, caused by the severe acute respiratory syndrome coronavirus 2 (SARS-CoV-2), millions of lives have been lost, posing formidable challenges to healthcare systems worldwide. Our study aims to conduct a meta-analysis to evaluate the efficacy of vitamin C supplementation in reducing in-hospital mortality rates and shortening the length of ICU or hospital stays among patients diagnosed with COVID-19.

**Methods:**

A comprehensive systematic review and meta-analysis was conducted, sourcing data from PubMed, Embase, Scopus, and the Cochrane Central Register of Controlled Trials. Our analysis focused on randomized clinical trials comparing the efficacy of vitamin C supplementation with standard care in adult COVID-19 patients.

**Results:**

Through meticulous examination of 11 clinical trials, our meta-analysis found that vitamin C supplementation did not reduce in-hospital mortality rates in COVID-19 patients compared to those receiving standard care (Risk Ratio [RR] = 0.85; 95% Confidence Interval [CI]: 0.62–1.17; *p* = 0.31). Similarly, the analysis indicated no significant difference in the length of ICU stays between both cohorts. Additionally, the occurrence of other adverse events was found to be similar across both groups treated with vitamin C supplementation and standard care (all *p* > 0.05).

**Conclusion:**

Vitamin C supplementation did not reduce in-hospital mortality or ICU stay durations in patients with COVID-19. The interpretation of these findings is limited by the small number of available studies and participants, which affects the strength of the conclusions.

**Clinical trial registration:**

Identifier CRD42024497474.

## 1 Introduction

The COVID-19 pandemic, declared in March 2020 by the World Health Organization, has brought unprecedented challenges, with over 200 million confirmed cases and 4.25 million fatalities globally as of November 22, 2021 (1). Despite advances in immunomodulatory and antiviral therapies, their efficacy varies, and global access remains unequal (2).

Vitamin C, previously proposed as a potential therapy for infections before the advent of COVID-19 (3), has a theoretical basis for its use in treating infections. However, clinical study results regarding vitamin C supplementation have been inconsistent. At the onset of the pandemic, the World Health Organization emphasized the potential of vitamin C as an immunomodulatory agent (4). Considering humans cannot synthesize vitamin C and many COVID-19 patients exhibit low levels, supplementation could theoretically offer benefits.

The recent increase in randomized clinical trials (RCTs) examining the impact of vitamin C on patients with COVID-19 has garnered significant attention. Earlier systematic reviews suggested that vitamin C reduced hospital mortality in patients with COVID-19 (5). However, those meta-analysis faced limitations due to its small size and inclusion of non-randomized trials. Most notably, two consecutive randomized controlled trials (RCTs) (6), the largest studies on this subject, failed to replicate the beneficial effects of vitamin C on mortality in COVID-19 patients. Amidst these uncertainties and conflicts, there is a critical need for a comprehensive assessment of the evidence to elucidate the true impact of vitamin C supplementation on outcomes in COVID-19 patients.

This meta-analysis aims to address these gaps by rigorously evaluating the existing RCTs to determine the efficacy of vitamin C supplementation in reducing in-hospital mortality rates and shortening ICU or hospital stays among COVID-19 patients. By synthesizing the available evidence, we seek to provide clarity on the role of vitamin C in the management of COVID-19 and guide future research and clinical practice.

## 2 Methods

### 2.1 Protocol and guidance

This study diligently followed the stringent guidelines set forth by the Preferred Reporting Items for Systematic Reviews and Meta-Analyses (PRISMA), a universally recognized framework that promotes transparent and thorough reporting in systematic reviews and meta-analyses (7, 8). Furthermore, demonstrating our dedication to methodological precision, we proactively registered our research protocol with the International Prospective Register of Systematic Reviews (PROSPERO), receiving the unique registration number CRD42024497474. This proactive pre-registration aimed explicitly at fostering transparency, showcasing our commitment to upholding the highest standards in conducting systematic reviews and meta-analyses.

### 2.2 Inclusion criteria

1. Population: Studies involving individuals aged 18 years and above diagnosed with COVID-19.
2. Intervention: Our inclusion criteria encompassed studies evaluating the effects of vitamin C supplementation, whether administered as a standalone treatment or as part of combination therapy.
3. Comparison intervention: Studies comparing the effects of vitamin C supplementation with standard treatment.
4. Outcome: The primary aim of this study is to assess the influence of vitamin C supplementation on in-hospital mortality rates among patients diagnosed with COVID-19, comparing its effects against those receiving standard conventional treatment. Additionally, secondary outcomes encompass examining the impact of vitamin C supplementation on the duration of ICU or hospital stays in COVID-19 patients.
5. Study design: We included only randomized controlled trials (RCTs).

### 2.3 Exclusion criteria

Non-RCT studies, including retrospective studies and crossover trials, were excluded from this review to ensure the robustness of the results and avoid potential biases such as carryover effects.

### 2.4 Information sources and search strategy

A thorough systematic search was executed across various databases, notably the Cochrane Central Register of Controlled Trials, PubMed, Embase and Scopus. The search encompassed articles from the inception of the databases up until December 28, 2023. Language restrictions were not applied during the search process. The complete search strategy can be found in Supplementary Table S1.

### 2.5 Study selection

The systematic search strategy was conducted to identify relevant articles. Two independent reviewers (WX and YL) screened the titles and abstracts of the retrieved studies. Subsequently, the selected articles were thoroughly assessed in full-text format by the same reviewers. Discrepancies or uncertainties encountered during the review process were resolved through collaborative discussion, with a third reviewer available to serve as an arbitrator if needed.

### 2.6 Data extraction

Two independent reviewers (WX and YL) performed data extraction from the included trials. The extraction process focused on gathering information pertaining to the study population, number of participants, mean age, and intervention details. To ensure the accuracy and reliability of the extracted data, a cross-check was conducted by a third reviewer to identify any errors or discrepancies. In instances where discrepancies arose between the two initial reviewers, a consensus was reached through a discussion among all reviewers.

### 2.7 Assessment of risk of bias

In the methodological assessment of the included trials, two independent reviewers (YL and WX) utilized the Cochrane Risk of Bias tool to evaluate the potential bias (9). Our analysis utilized a tool that assesses bias across seven distinct domains, assigning each trial a study-level score indicative of the risk of bias (low, high, or unclear) within each domain. Any disagreements between reviewers were addressed through detailed discussions. In instances where consensus remained elusive, a conclusive determination was made by a third author (YZ).

### 2.8 Confidence of evidence

Two evaluators, (YT and WX), independently appraised the quality of evidence pertaining to both primary and secondary outcomes employing the Grading of Recommendation, Assessment, Development, and Evaluation (GRADE) framework. Subsequently, they classified the gathered evidence into four distinct tiers: high, moderate, low, or very low. This classification was grounded on an array of factors, including the design of the study, the risk of bias, variability in outcomes, the accuracy of the data, and the relevance of the trials reviewed.

### 2.9 Data analysis

Statistical evaluations were carried out utilizing RevMan software (version 5.3), furnished by The Cochrane Collaboration. For outcomes measured in a binary manner, we calculated the relative risk (RR) accompanied by 95% confidence intervals (CI) (10). A *p*-value below 0.05 was considered statistically significant, and the I2 test was employed to quantify the proportion of total variability attributable to heterogeneity, providing a measure of the degree of heterogeneity. This helps us ascertain whether the studies are sufficiently homogeneous to justify pooling their results. Random-effects models were applied in cases where I2 exceeded 50%, indicating substantial heterogeneity, whereas fixed-effects models were employed when I2 was below 50%, to enhance the reliability of the findings. By adopting these rigorous statistical methods and principles, we aimed to provide a comprehensive and robust analysis of the available data. The potential for small study effects was qualitatively evaluated through the visual inspection of the funnel plot. Additionally, quantitative analysis was conducted using the Egger test, Peters test, and Harbord test. This comprehensive approach allowed for a thorough evaluation of any potential bias caused by small study effects.

### 2.10 Subgroup analysis

Subgroup analysis was conducted to assess the in-hospital mortality among critically ill patients. The analysis aimed to investigate potential differences in outcomes across various sub-groups.

### 2.11 Sensitivity analysis

We conducted a sensitivity analysis using the following methods: (1) Excluding studies with high or unknown risk of bias; (2) excluding trials with a weight less than 10%.

### 2.12 Trial sequential analysis

To reduce the risk of type I error, we performed a trial sequential analysis (TSA 0.9Beta), combining the estimated information size with a revised significance threshold. This approach aimed to maintain a 5% type I error risk and attain an 80% statistical power, using a two-sided trial sequential analysis.

## 3 Results

### 3.1 Included studies and study characteristics

**Figure 1:**
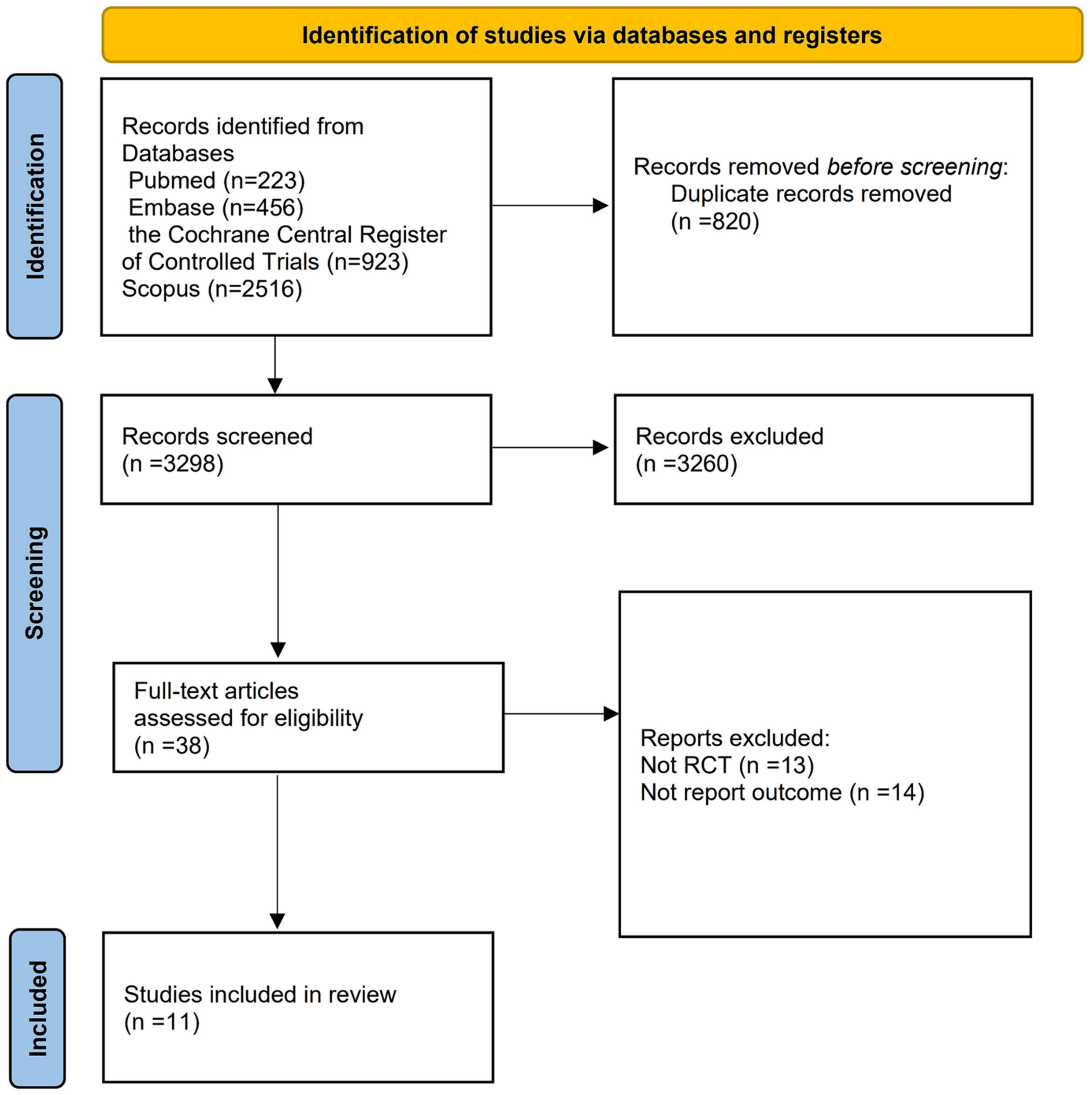
Search strategy and final included and excluded studies.

The initial search strategy yielded 4,118 records. After removing duplicate entries, we were left with 3,298 unique records. These records were then carefully assessed through the examination of their titles, abstracts, and full texts. Through this thorough evaluation process, we identified 11 trials that satisfied the detailed criteria established for this systematic review. Please refer to Figure 1 for a visual representation of this process.

Table 1 provides an overview of the trial characteristics considered in this study. The identified trials were published between 2020 and 2023, featuring sample sizes that varied from 20 to 2,591 patients.

**Table 1:** Characteristics of included studies.

Study	Country	Patients, *n*	Age, years	Male, (%)	Treatment in the intervention group	Treatment in the control group
Beigmohammadi et al. (11)	Iran	60	51.00 *±* 17.25	15 (50.0%)	25,000 IU daily of vitamins A, 600,000 IU once during the study of D, 300 IU twice daily of E, 500 mg four times daily of C, and one amp daily of B complex	Standard of care alone
Coppock et al. (12)	United States	66	60 *±* 17	22 (50.0%)	Escalating doses of intravenous vitamin C plus standard of care	Standard of care alone
Darban et al. (13)	Iran	20	NR	NR	Standard care plus intravenous vitamin C (2 g, q6hr), oral melatonin (6 mg, q6hr), and oral zinc sulfate (50 mg, q6hr)	Standard of care alone
Hakamifard et al. (20)	Iran	72	35.68	24 (63.2%)	Oral vitamin C 1000 mg daily plus oral vitamin E 400 IU daily in addition to the national standard treatment regimen	Standard regimen alone
Kumari et al. (15)	Pakistan	150	52 *±* 11	NR	50 mg/kg/day of intravenous vitamin C	Standard therapy
Majidi et al. (16)	Iran	100	59.42 *±* 15.07	19	One capsule of 500 mg of vitamin C daily	The same nutrition except for vitamin C supplements
Adhikari et al. (6)	Canada	2,591	59.97 ± 3.08	655 (63.2%)	Vitamin C administered intravenously (50 mg/kg of body weight administered intravenously over 30–60 min every 6 h)	Placebo or no vitamin C
JamaliMoghadamSiahkali et al. (14)	Iran	60	57.53 *±* 18.27	15	High-dose intravenous vitamin C (6 g daily)	Lopinavir/ritonavir and hydroxychloroquine
Tehrani et al. (17)	Iran	44	58 *±* 19	8	Intravenous vitamin C at a dose of 2 g every 6 h	Standard therapy
Thomas et al. (18)	USA	98	45.6 *±* 15.0	15	10 days of vitamin C (8,000 mg)	Standard therapy
Zhang et al. (19)	China	56	66.3 *±* 11.2	15 (55.6%)	12 g of vitamin C/50 mL every 12 h	Placebo

NR, not reported.

### 3.2 Risk-of-bias assessments

Risk-of-bias assessments are detailed in Supplementary Figures S1, S2. Among the evaluated trials, five were found to have a low risk of bias, three presented an unclear risk, and three exhibited a high risk. The quality of evidence for the primary outcome, as appraised using the GRADE methodology, ranged from moderate to high, as shown in Table 2.

**Table 2:** Summary of findings and strength of evidence.

Outcome	NO. of patients (trials)	Relative effect (95% CI)	Absolute effect estimates (per 1,000)			Quality of the evidence
			Vitamin-C	Control	Difference	
**The primary outcome**						
In-hospital mortality	3,244 (10)	RR 0.85 [0.62, 1.17]	538	386	−27 [−67, 30]	Moderate^#^
**The secondary outcomes**						
Intensive care unit length of stay	136 (3)	MD 0.86 [−0.54, 2.25]	–	–	MD 0.86 [−0.54, 2.25]	Moderate*
Hospital length of stay	382 (5)	MD −0.54 [−3.10, 2.01]	–	–	MD −0.54 [−3.10, 2.01]	High

CI, confidence interval; MD, mean difference; RR, risk ratio.

^# Inconsistency: due to high I2 value of 75%.^

^*^ Imprecise: due to small number of studies and few participants.

### 3.3 Outcomes

**Figure 2:**
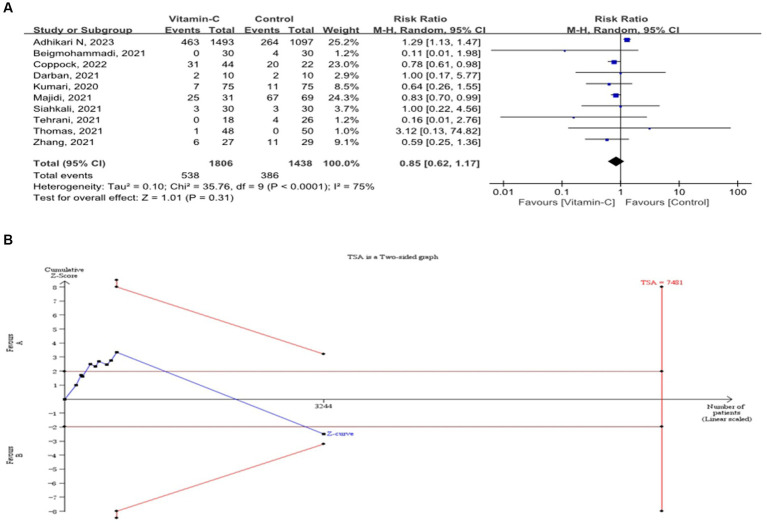
Forest plots of in-hospital mortality among COVID-19 patients with and without vitamin C supplementation and trial sequential analysis. **(A)** Forest plot for in-hospital mortality among COVID-19 patients with and without vitamin C. **(B)** Trial sequential analysis for in-hospital mortality among COVID-19 patients with and without vitamin C.

This meta-analysis revealed that the overall in-hospital mortality rate was comparable between COVID-19 patients supplemented with vitamin C and those undergoing standard treatment (RR = 0.85; 95% CI: 0.62–1.17) (Figure 2). Furthermore, the trial sequential analysis of mortality indicated a significant shortfall in reaching the required information size (Figure 2). In light of the conducted sensitivity analysis, our findings remain robust, underscoring the reliability of our results (Supplementary Table S2). Moreover, according to the results of the symmetric funnel plot analysis, it is apparent that the funnel plot exhibited asymmetry, thereby underscoring the imperative for quantitative analysis (Supplementary Figure S3).

Subgroup analysis, specifically targeting mortality outcomes among critically ill patients, was conducted. However, this analysis did not reveal any statistically significant differences across any of the outcomes or sub-groups examined (Supplementary Figure S4).

**Figure 3:**
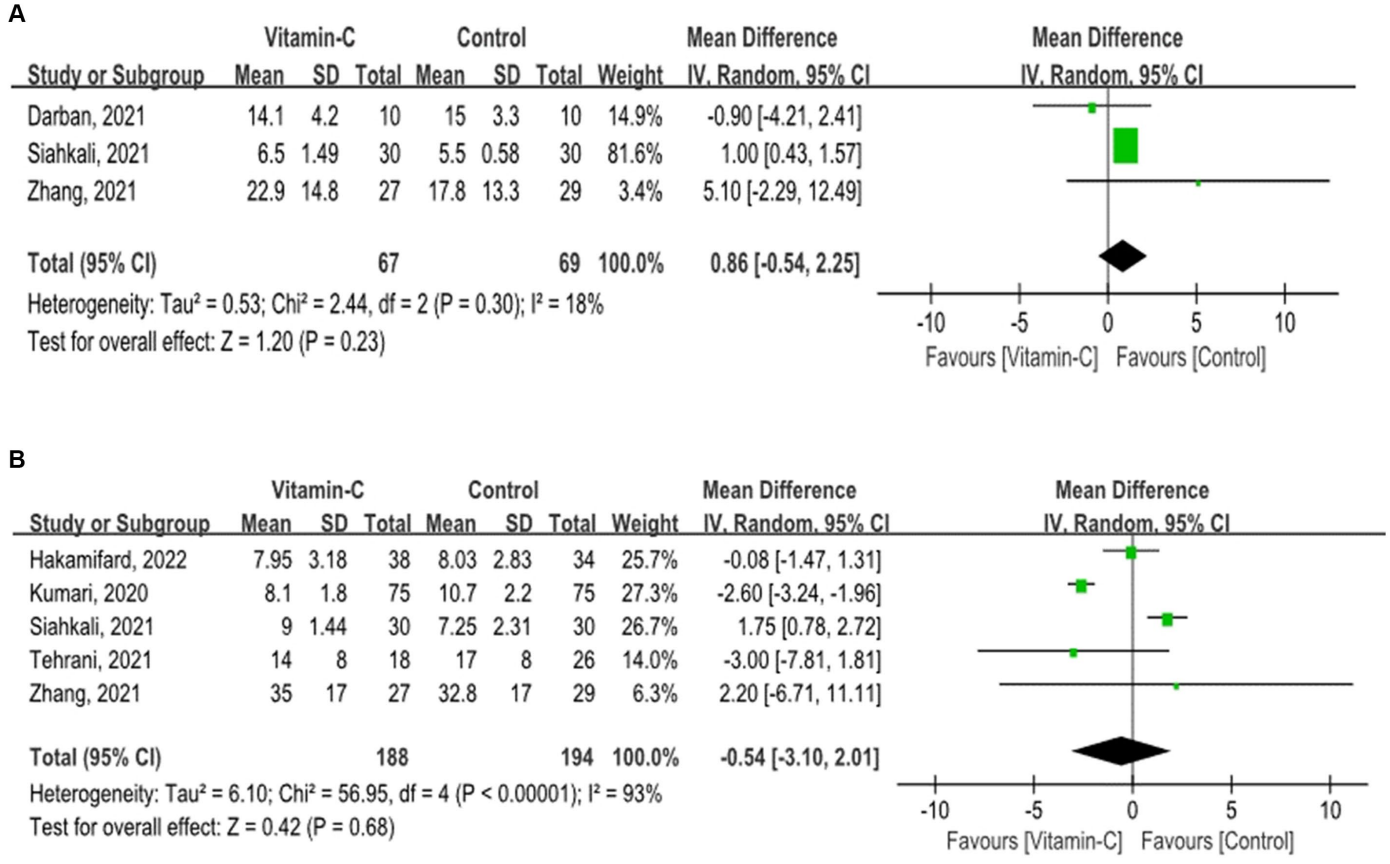
Forest plots for secondary outcomes. **(A)** Forest plot of Intensive Care Unit Length of stay among COVID-19 patients with and without vitamin C supplementation. **(B)** Forest plot of hospital length of stay among COVID-19 patients with and without vitamin C supplementation.

Patients supplemented with vitamin C did not exhibit a prolonged ICU stay (OR = 0.86; 95% CI: −0.54 to 2.25; *p* = 0.23) or hospital stay (OR = −0.54; 95% CI: −3.10 to 2.01; *p* = 0.68) (Figure 3), with a comparable duration to those undergoing standard treatment.

## 4 Discussion

In our meta-analysis of 11 RCTs (6, 11–20), we found that vitamin C supplementation did not reduce in-hospital mortality among COVID-19 patients.

This finding contrasted with earlier meta-analyses and systematic reviews (5, 21). Kow et al. suggested a survival benefit for vitamin C in patients with severe COVID-19, and Olczak-Prucet al. found a reduction in hospital mortality due to vitamin C use. These studies, however, acknowledged the limitations of their evidence, including small sample sizes and methodological challenges. Our analysis incorporates a recent large-scale trial, significantly increasing our patient sample size and enhancing the statistical power of our findings. This inclusion challenges earlier suggestions of vitamin C reducing COVID-19 hospital mortality and advises healthcare providers and policymakers to consider our more comprehensive evidence.

Our review’s strengths lie in its rigorous approach, including a thorough evidence search, adherence to a predefined protocol, and meticulous quality assessment by multiple reviewers. By focusing exclusively on RCTs, we have elevated the quality of evidence, reinforcing the reliability of our conclusions.

This study is subject to several limitations. First, the number of included studies and the overall available data were limited, which affects the robustness and generalizability of the findings. Additionally, there was considerable variability in both the dosage and duration of vitamin C treatment across the trials, as well as evolving standards of COVID-19 care, which may have introduced potential biases. The data available for conducting subgroup analyses, particularly regarding comorbidities such as obesity, diabetes, hypertension, and kidney disease, were also insufficient. Furthermore, challenges related to participant engagement and data collection during the pandemic posed additional difficulties in the interpretation of the results.

## 5 Conclusion

This meta-analysis challenges the efficacy of vitamin C supplementation in decreasing in-hospital mortality among COVID-19 patients. Emerging data suggest a neutral impact on mortality rates and ICU durations, highlighting the evolving landscape of COVID-19 treatment research. However, the limited availability of published data that could be integrated into the meta-analysis impacts the strength of our conclusions. As such, further large-scale randomized controlled trials are essential to provide more definitive evidence and guide future clinical protocols.

## Data Availability

The original contributions presented in the study are included in the article/Supplementary material, further inquiries can be directed to the corresponding author.

## References

[1] Yang B Xu LY Li LY Qiao DF Du SH Yue X . Pathological changes and cause of death associated with the global novel coronavirus disease (COVID-19). Fa Yi Xue Za Zhi. (2023) 39:586–95. doi: 10.12116/j.issn.1004-5619.2023.430703, PMID: 38228478

[2] Ezzikouri S Nourlil J Benjelloun S Kohara M Tsukiyama-Kohara K. Coronavirus disease 2019-historical context, virology, pathogenesis, immunotherapy, and vaccine development. Hum Vaccin Immunother. (2020) 16:2992–3000. doi: 10.1080/21645515.2020.1787068, PMID: 32755425 PMC8641599

[3] Al-Obaidi ZMJ Hussain YA Ali AA Al-Rekabi MD. The influence of vitamin-C intake on blood glucose measurements in COVID-19 pandemic. J Infect Dev Ctries. (2021) 15:209–13. doi: 10.3855/jidc.13960, PMID: 33690202

[4] Vollbracht C Kraft K. Feasibility of vitamin C in the treatment of post viral fatigue with focus on long COVID, based on a systematic review of IV vitamin C on fatigue. Nutrients. (2021) 13:1154. doi: 10.3390/nu13041154 33807280 PMC8066596

[5] Olczak-Pruc M Swieczkowski D Ladny JR Pruc M Juarez-Vela R Rafique Z . Vitamin C supplementation for the treatment of COVID-19: a systematic review and Meta-analysis. Nutrients. (2022) 14:4217. doi: 10.3390/nu14194217 36235869 PMC9570769

[6] Adhikari NKJ Hashmi M Vijayaraghavan BKT Haniffa R Beane A Webb SA . Intravenous vitamin C for patients hospitalized with COVID-19: two harmonized randomized clinical trials. JAMA. (2023) 330:1745–59. doi: 10.1001/jama.2023.21407, PMID: 37877585 PMC10600726

[7] Page MJ McKenzie JE Bossuyt PM Boutron I Hoffmann TC Mulrow CD . The PRISMA 2020 statement: an updated guideline for reporting systematic reviews. BMJ. (2021) 372:n71. doi: 10.1136/bmj.n71 33782057 PMC8005924

[8] Hutton B Salanti G Caldwell DM Chaimani A Schmid CH Cameron C . The PRISMA extension statement for reporting of systematic reviews incorporating network meta-analyses of health care interventions: checklist and explanations. Ann Intern Med. (2015) 162:777–84. doi: 10.7326/M14-2385, PMID: 26030634

[9] Sterne JAC Savović J Page MJ Elbers RG Blencowe NS Boutron I . RoB 2: a revised tool for assessing risk of bias in randomised trials. BMJ. (2019) 366:l4898. doi: 10.1136/bmj.l4898 31462531

[10] Warn DE Thompson SG Spiegelhalter DJ. Bayesian random effects meta-analysis of trials with binary outcomes: methods for the absolute risk difference and relative risk scales. Stat Med. (2002) 21:1601–23. doi: 10.1002/sim.1189, PMID: 12111922

[11] Beigmohammadi MT Bitarafan S Hoseindokht A Abdollahi A Amoozadeh L Soltani D. The effect of supplementation with vitamins a, B, C, D, and E on disease severity and inflammatory responses in patients with COVID-19: a randomized clinical trial. Trials. (2021) 22:802. doi: 10.1186/s13063-021-05795-4, PMID: 34776002 PMC8590866

[12] Coppock D Violet PC Vasquez G Belden K Foster M Mullin B . Pharmacologic ascorbic acid as early therapy for hospitalized patients with COVID-19: a randomized clinical trial. Life (Basel, Switzerland). (2022) 12:453. doi: 10.3390/life12030453 35330204 PMC8954118

[13] Darban M Malek F Memarian M Gohari A Kiani A Emadi A . Efficacy of high dose vitamin C, melatonin and zinc in Iranian patients with acute respiratory syndrome due to coronavirus infection: a pilot randomized trial. J Cell Mol Anesth. (2021) 6:164–7. doi: 10.22037/jcma.v6i2.32182

[14] JamaliMoghadamSiahkali S Zarezade B Koolaji S SeyedAlinaghi SA Zendehdel A Tabarestani M . Safety and effectiveness of high-dose vitamin C in patients with COVID-19: a randomized open-label clinical trial. Eur J Med Res. (2021) 26:20. doi: 10.1186/s40001-021-00490-1, PMID: 33573699 PMC7877333

[15] Kumari P Dembra S Dembra P Bhawna F Gul A Ali B . The role of vitamin C as adjuvant therapy in COVID-19. Cureus. (2020) 12:e11779. doi: 10.7759/cureus.11779 33409026 PMC7779177

[16] Majidi N Rabbani F Gholami S Gholamalizadeh M BourBour F Rastgoo S . The effect of vitamin C on pathological parameters and survival duration of critically ill coronavirus disease 2019 patients: a randomized clinical trial. Front Immunol. (2021) 12:717816. doi: 10.3389/fimmu.2021.717816 34975830 PMC8714637

[17] Tehrani S Yadegarynia D Abrishami A Moradi H Gharaei B Rauofi M . An investigation into the effects of intravenous vitamin C on pulmonary CT findings and clinical outcomes of patients with COVID 19 pneumonia a randomized clinical trial. Urol J. (2022) 19:460–5. doi: 10.22037/uj.v18i.6863, PMID: 34746999

[18] Thomas S Patel D Bittel B Wolski K Wang Q Kumar A . Effect of high-dose zinc and ascorbic acid supplementation vs usual care on symptom length and reduction among ambulatory patients with SARS-CoV-2 infection: the COVID a to Z randomized clinical trial. JAMA Netw Open. (2021) 4:e210369. doi: 10.1001/jamanetworkopen.2021.0369, PMID: 33576820 PMC7881357

[19] Zhang J Rao X Li Y Zhu Y Liu F Guo G . Pilot trial of high-dose vitamin C in critically ill COVID-19 patients. Ann Intensive Care. (2021) 11:5. doi: 10.1186/s13613-020-00792-3 33420963 PMC7794643

[20] Hakamifard A Soltani R Maghsoudi A Rismanbaf A Aalinezhad M Tarrahi MJ . The effect of vitamin E and vitamin C in patients with COVID-19 pneumonia; a randomized controlled clinical trial. Immunopathol Persa. (2022) 8:e8. doi: 10.34172/ipp.2022.08

[21] Kow CS Hasan SS Ramachandram DS. The effect of vitamin C on the risk of mortality in patients with COVID-19: a systematic review and meta-analysis of randomized controlled trials. Inflammopharmacology. (2023) 31:3357–62. doi: 10.1007/s10787-023-01200-5, PMID: 37071316 PMC10111321

