# Supplementary material for: Effect of vitamin C supplementation on outcomes in patients with COVID-19: a systematic review and meta-analysis
Source: Front Nutr. 2024 Oct 3;11:1465670. doi: 10.3389/fnut.2024.1465670 (PMC11484096; doi:10.3389/fnut.2024.1465670)

**Supplemental e-material**

| Table S1: Search strategy |
| --- |
| Table S2: Sensitivity analysis of meta-analysis |
| Figure S1: Risk of bias summary |
| Figure S2: Risk of bias graph |
| Figure S3: Publication bias plot |
| Figure S4: The subgroup analysis for primary outcome |

**S1 Table. Search strategy**

| **PubMed** | | |
| --- | --- | --- |
|  | "Ascorbic Acid"[Mesh] | 45789 |
|  | "COVID-19"[Mesh] | 37439 |
|  | Acid, Ascorbic[Title/Abstract] OR L-Ascorbic Acid[Title/Abstract] OR Acid.L-Ascorbic[Title/Abstract] OR LAscorbic Acid[Title/Abstract] OR Vitamin C[Title/Abstract] OR Hybrin[Title/Abstract] OR Magnorbin[Title/Abstract] OR Sodium Ascorbate[Title/Abstract] OR Ascorbate.Sodium[Title/Abstract] OR Ascorbic Acid,Monosodium Salt[Title/Abstract] OR Ferrous Ascorbate[Title/Abstract] OR Ascorbate.Ferrous[Title/Abstract] OR Maanesium Ascorbate[Title/Abstract] OR Ascorbate,Magnesium[Title/Abstract] OR Magnesium di-L-Ascorbate[Title/Abstract] OR Magnesium di L Ascorbate[Title/Abstract] OR di-I -Ascorbate.Magnesium[Title/Abstract] OR Magnesium Ascorbicum[Title/Abstract] | 49079 |
|  | COVID 19[Title/Abstract] OR 2019-nCoV Infection[Title/Abstract] OR 2019 nCoV Infection[Title/Abstract] OR 2019-nCoV Infections[Title/Abstract] OR Infection,2019-nCoV[Title/Abstract] OR SARS-CoV-2 Infection[Title/Abstract] OR Infection.SARS-COV-2[Title/Abstract] OR SARS CoV 2 Infection[Title/Abstract] OR SARS-CoV-2 Infections[Title/Abstract] OR 2019 Novel Coronavirus Disease[Title/Abstract] OR 2019 Novel Coronavirus Infection[Title/Abstract] OR COVID-19 Virus Infection[Title/Abstract] OR COVID 19 Virus Infection[Title/Abstract] OR COVID-19 Virus Infections[Title/Abstract] OR Infection.COVID-19 Virus[Title/Abstract] OR Virus Infection,COVID-19[Title/Abstract] OR COVID19[Title/Abstract] OR Coronavirus Disease 2019[Title/Abstract] OR Disease 2019.Coronavirus[Title/Abstract] OR Coronavirus Disease-19[Title/Abstract] OR Coronavirus Disease 19[Title/Abstract] OR Severe Acute Respiratory Severe Acute Respiratory[Title/Abstract] OR COVID-19 Virus Disease[Title/Abstract] OR COVID 19 Virus Disease[Title/Abstract] OR COVID-19 Virus Diseases[Title/Abstract] OR Disease,COVID-19 Virus[Title/Abstract] OR Virus Disease,COVID-19[Title/Abstract] OR SARS Coronavirus 2 Infection[Title/Abstract] OR 2019-nCoV Disease[Title/Abstract] OR 2019 nCoV Disease[Title/Abstract] OR 2019-nCoV Diseases[Title/Abstract] OR Disease,2019-nCoV[Title/Abstract] OR COVID-19 Pandemic[Title/Abstract] OR COVID 19 Pandemic[Title/Abstract] OR Pandemic,COVID-19[Title/Abstract] OR COVID-19 Pandemics[Title/Abstract] | 376909 |
|  | vitamin C[Title/Abstract] OR ascorbic acid[Title/Abstract] OR VC[Title/Abstract] OR L-ascorbic acid[Title/Abstract] OR intravenous infusion[Title/Abstract] OR intravenous drip[Title/Abstract] OR ascorbic acid[Title/Abstract] OR infusions, intravenous[Title/Abstract] | 98394 |
|  | #1 OR #3 OR #5 | 123029 |
|  | 2019-nCoV[Title/Abstract] OR coronavirus disease 2019[Title/Abstract] OR COVID-19[Title/Abstract] OR SARS-CoV-2[Title/Abstract] OR novel coronavirus[Title/Abstract] OR COVID-19[Title/Abstract] OR coronavirus disease 2019[Title/Abstract] OR 2019-nCoV[Title/Abstract] OR novel coronavirus[Title/Abstract] | 384654 |
|  | #2 OR #4 OR #7 | 402719 |
|  | #6 AND #8 | 849 |
|  | animals | 7492265 |
|  | #9 NOT #10 | 807 |
|  | Randomized Controlled Trials as Topic OR randomized controlled trial OR controlled clinical trial OR randomized OR randomly OR trial | 2800701 |
|  | #11 AND #12 | 222 |
| **EMBASE** | | |
| 1. | 'ascorbic acid'/exp | 119811 |
| 2. | 'coronavirus disease 2019'/exp | 372613 |
| 3. | ('acid, ascorbic' OR 'l-ascorbic acid' OR 'acid.l-ascorbic' OR lascorbic) AND acid OR 'vitamin c' OR hybrin OR magnorbin OR 'sodium ascorbate' OR 'ascorbate.sodium' OR 'ascorbic acid,monosodium salt' OR 'ferrous ascorbate' OR 'ascorbate.ferrous' OR 'maanesium ascorbate' OR 'ascorbate,magnesium' OR 'magnesium di-l-ascorbate' OR 'magnesium di l ascorbate' OR 'di-i -ascorbate.magnesium' OR 'magnesium ascorbicum':ab,ti | 40977 |
| 4. | 'covid 19' OR '2019 ncov infection' OR '2019-ncov infection' OR 'infection,2019-ncov' OR 'sars-cov-2 infection' OR 'infection.sars-cov-2' OR 'sars cov 2 infection' OR 'sars-cov-2 infections' OR '2019 novel coronavirus disease' OR '2019 novel coronavirus infection' OR 'covid-19 virus infection' OR 'covid 19 virus infection' OR 'covid-19 virus infections' OR 'infection.covid-19 virus' OR 'virus infection,covid-19' OR covid19 OR 'coronavirus disease 2019' OR 'disease 2019.coronavirus' OR 'coronavirus disease-19' OR 'coronavirus disease 19' OR 'severe acute respiratory severe acute respiratory' OR 'covid-19 virus disease' OR 'covid 19 virus disease' OR 'covid-19 virus diseases' OR 'disease,covid-19 virus' OR 'virus disease,covid-19' OR 'sars coronavirus 2 infection' OR '2019-ncov disease' OR '2019 ncov disease' OR '2019-ncov diseases' OR 'disease,2019-ncov' OR 'covid-19 pandemic' OR 'covid 19 pandemic' OR 'pandemic,covid-19' OR 'covid-19 pandemics':ab,ti | 457001 |
| 5. | 'vitamin c' OR 'vc or l-ascorbic acid' OR 'intravenous infusion' OR 'intravenous drip' OR 'ascorbic acid' OR 'infusions, intravenous':ab,ti | 165482 |
| 6. | 'sars-cov-2' OR 'novel coronavirus' OR 'covid-19' OR 'coronavirus disease 2019' OR '2019-ncov' OR 'novel coronavirus':ab,ti | 473791 |
| 7. | 'randomized controlled trials as topic' OR 'randomized controlled trial' OR 'controlled clinical trial' OR randomized OR randomly OR trial | 3215040 |
| 8. | #1 OR #3 OR #5 | 166796 |
| 9. | #2 OR #4 OR #6 | 475012 |
| 10. | #7 AND #8 AND #9 | #7 AND #8 AND #9 |
| 11. | animals | 1024116 |
| 12. | #10 NOT #11 | 455 |
| **COCHRANE CENTRAL** | | |
| 1. | MeSH descriptor: [Ascorbic Acid] explode all trees | 2606 |
| 2. | MeSH descriptor: [COVID-19] explode all trees | 4985 |
| 3. | Acid, Ascorbic OR L-Ascorbic Acid OR Acid.L-Ascorbic OR LAscorbic Acid OR Vitamin C OR Hybrin OR Magnorbin OR Sodium Ascorbate OR Ascorbate.Sodium OR Ascorbic Acid,Monosodium Salt OR Ferrous Ascorbate OR Ascorbate.Ferrous OR Maanesium Ascorbate OR Ascorbate,Magnesium OR Magnesium di-L-Ascorbate OR Magnesium di L Ascorbate OR Magnesium Ascorbicum | 15298 |
| 4. | MeSH descriptor: [SARS-CoV-2] explode all trees | 2458 |
| 5. | coronavirus disease 2019 | 8019 |
| 6. | SARS-CoV-2 | 589 |
| 7. | COVID-19 | 18054 |
| 8. | SARS-CoV-2 Infection OR Infection.SARS-COV-2 OR SARS CoV 2 Infection OR SARS-CoV-2 Infections OR 2019 Novel Coronavirus Disease OR 2019 Novel Coronavirus Infection OR COVID-19 Virus Infection OR COVID 19 Virus Infection OR COVID-19 Virus Infections OR Infection.COVID-19 Virus | 18203 |
| 9. | Virus Infection,COVID-19 OR COVID19 OR Coronavirus Disease 2019 OR Disease 2019.Coronavirus OR Coronavirus Disease-19 OR Coronavirus Disease 19 OR Severe Acute Respiratory Severe Acute Respiratory OR COVID-19 Virus Disease OR COVID 19 Virus Disease OR COVID-19 Virus Diseases OR Disease,COVID-19 Virus OR Virus Disease,COVID-19 OR SARS Coronavirus 2 Infection | 23906 |
| 10. | vitamin C OR ascorbic acid OR VC OR L-ascorbic acid OR intravenous infusion OR intravenous drip OR ascorbic acid OR infusions, intravenous | 23932 |
| 11. | #2 OR #4 OR #5 OR #6 OR #7 OR #8 OR #9 | 56531 |
| 12. | #11 AND #12 | 1446 |
| 13. | Randomized Controlled Trials as Topic OR randomized controlled trial OR controlled clinical trial OR randomized OR randomly OR trial | 1649745 |
| 14. | #13 AND #14 | 1418 |
| 15. | animals | 26253 |
| 16. | #15 NOT #16 | 1129 |
| **SCOPUS** | | |
| 1 | ("ascorbic acid" OR "vitamin C" OR “Sodium Ascorbate" OR "L-ascorbic") AND ("coronavirus" OR "COVID 19" OR "COVID-19" OR "Corona" OR "COVID" OR "SARSCoV2") | 2156 |

Table S2: Sensitivity analysis of meta-analysis

|  | NO. patients(trials) | RR | 95%CI |
| --- | --- | --- | --- |
| All trials | 3244(10) | 0.85 | 0.62,1.17 |
| Excluding studies with high or unknown risk of bias | 2956(5) | 0.86 | 0.57,1.29 |
| Excluding trials with a weight less than 10% | 2756(3) | 0.95 | 0.63,1.41 |

Figure S1: Risk of bias summary


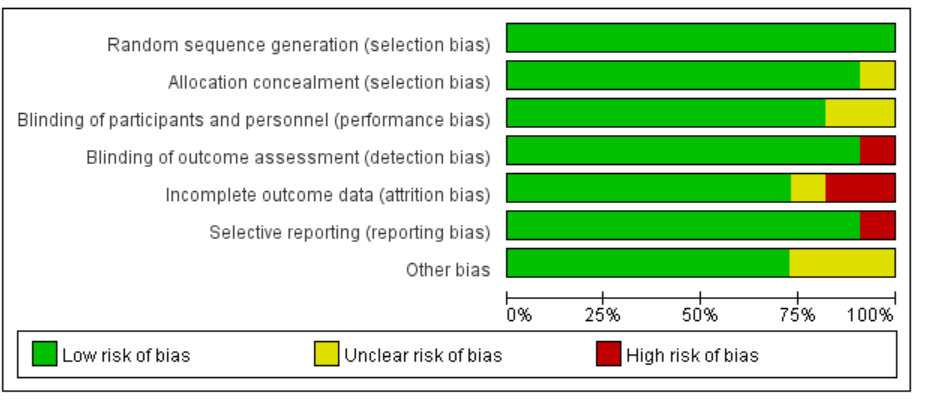


Figure S2: Risk of bias graph


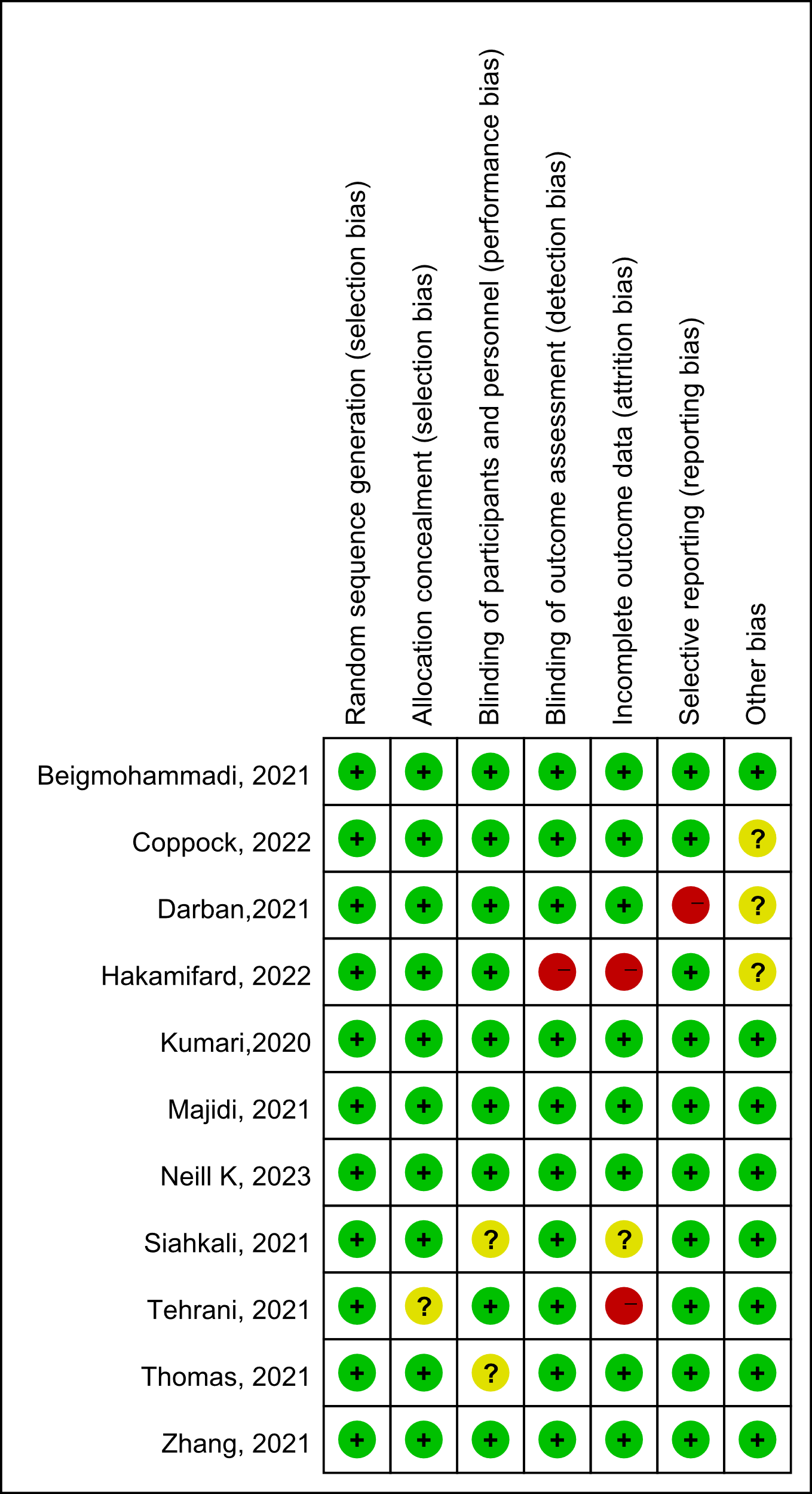


Figure S3: Publication bias plot
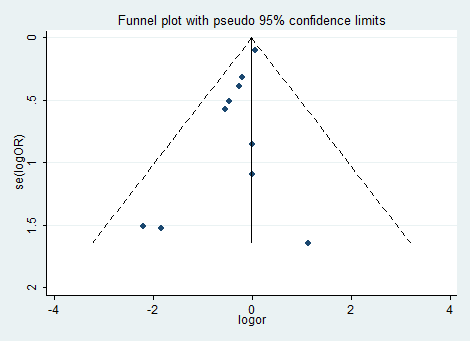


Figure S4: The subgroup analysis for primary outcome


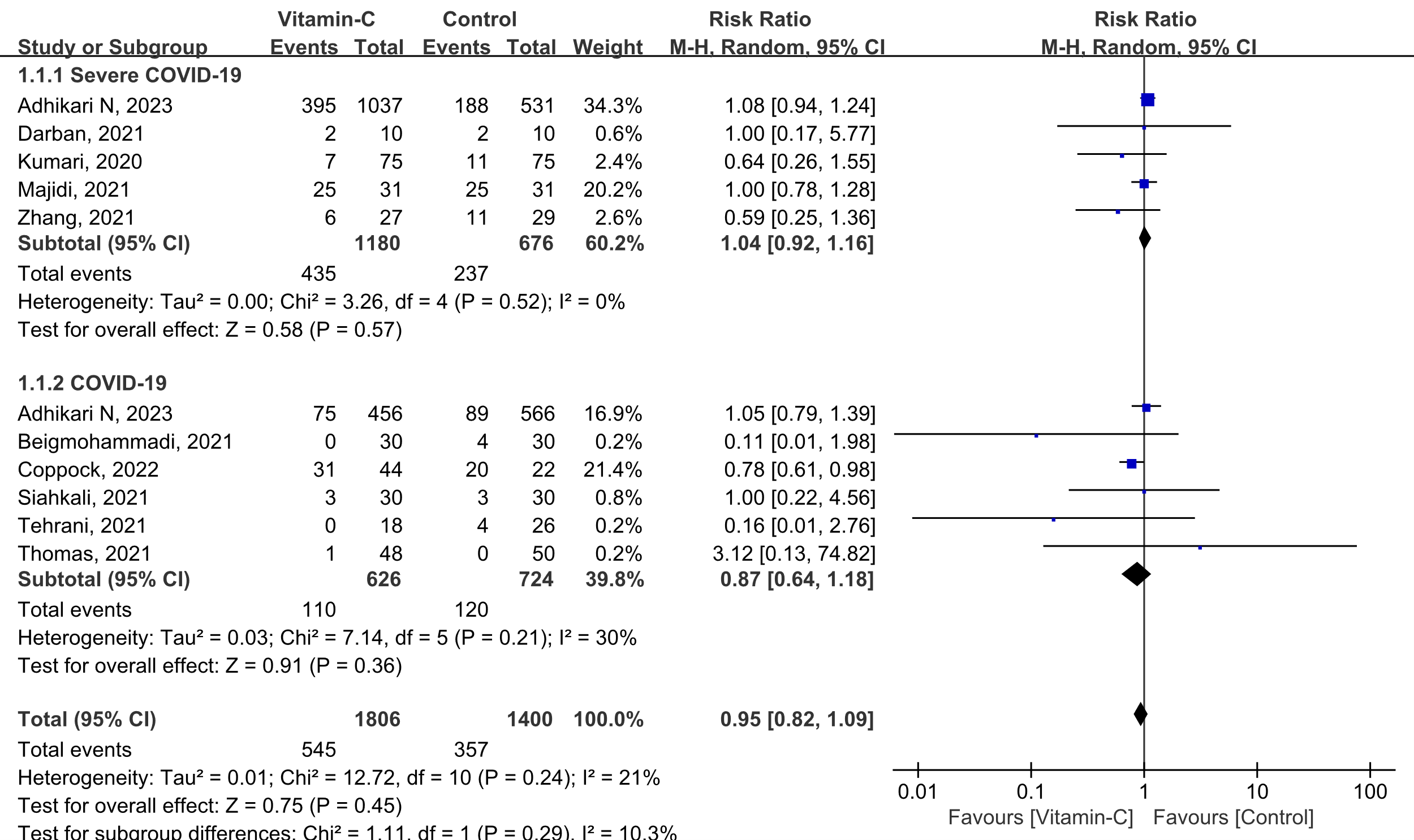

Supplement: Supplementary file 1 [file Data_Sheet_1.DOCX]
